# PrimedRPA: primer design for recombinase polymerase amplification assays

**DOI:** 10.1093/bioinformatics/bty701

**Published:** 2018-08-08

**Authors:** Matthew Higgins, Matt Ravenhall, Daniel Ward, Jody Phelan, Amy Ibrahim, Matthew S Forrest, Taane G Clark, Susana Campino

**Affiliations:** 1 Pathogen Molecular Biology Department, London School of Hygiene and Tropical Medicine (LSHTM), London, UK; 2 TwistDx, Coldhams Business Park, Cambridge, UK; 3 Department of Infectious Disease Epidemiology, LSHTM, London, UK

## Abstract

**Summary:**

Recombinase polymerase amplification (RPA), an isothermal nucleic acid amplification method, is enhancing our ability to detect a diverse array of pathogens, thereby assisting the diagnosis of infectious diseases and the detection of microorganisms in food and water. However, new bioinformatics tools are needed to automate and improve the design of the primers and probes sets to be used in RPA, particularly to account for the high genetic diversity of circulating pathogens and cross detection of genetically similar organisms. PrimedRPA is a python-based package that automates the creation and filtering of RPA primers and probe sets. It aligns several sequences to identify conserved targets, and filters regions that cross react with possible background organisms.

**Availability and implementation:**

PrimedRPA was implemented in Python 3 and supported on Linux and MacOS and is freely available from http://pathogenseq.lshtm.ac.uk/PrimedRPA.html.

**Supplementary information:**

Supplementary data are available at *Bioinformatics* online.

## 1 Introduction

The last decade has seen a prodigious increase in the development and adaptation of novel and existing isothermal amplification technologies for molecular diagnostics. Recombinase Polymerase Amplification (RPA) enables both sensitive and rapid isothermal DNA amplification (Piepenburg *et al.*, 2006). RPA is establishing itself as a robust alternative to PCR, and becoming a molecular tool of choice for the rapid, specific, and cost-effective identification of pathogens. Its minimal sample preparation requirements, low operation temperature (25–42°C), and commercial availability of freeze-dried reagents, mean this method has been applied in field laboratory settings and on-board automated sample-to-answer microfluidic devices. Further, this technique can be performed directly in non-processed samples, such as whole blood (Magro *et al.*, 2017).

There is no automated software for designing primer-probe sets for RPA. Identifying candidate regions for assay development can be difficult as regions need to be conserved, with little homology to potential background DNA. Also, the sequence for primers and a probe to bind should create as small an amplicon as possible. Any DNA in the reaction that is not the target can be considered as background. Existing primer design software such as *Primer3* (Untergasser *et al.*, 2012) and *RExPrimer* (Piriyapongsa *et al.*, 2009) cannot be used to automate TwistAmp^®^ exo probe design, as they are typically longer than what these programs allow and specific requirements need to be met, including the positioning of two thymidine residues in the probe to which the fluorophore and quencher are attached. To overcome these issues, we developed *Prime*r *d*esign for *RPA* (*PrimedRPA*), which automates the RPA primer and exo probe design process. In addition, as RPA is permissive to the presence of SNPs, the software can input and align several target sequences to account for the high genetic diversity of circulating pathogens. The software can also input several background sequences to avoid the design of primer/probes that can cross react with genetically similar organisms. Here we test the software against several pathogens and validate some of the resulting primers in the laboratory.

## 2 Materials and methods

**Fig. 1.**
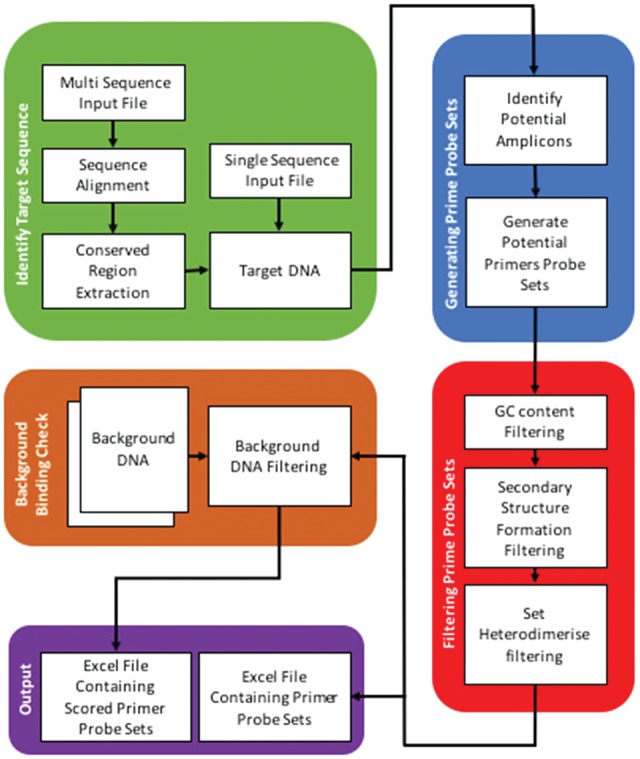
The analytical pipeline of *PrimedRPA*

The *PrimedRPA* package, developed in python, creates and filters RPA primer-probe sets specific for target DNA sequence(s). An overview of the package is presented in Figure 1. The user defines the input sequence(s), the parameters for filtering, and the sequence files for the background binding check, through altering the *PrimedRPA_Parameters.txt* file. The filtering parameters include primer, probe and amplicon lengths, GC content, ability to form a secondary structure and heterodimerise, and the tolerance of binding to background DNA (Fig. 1, Red and Brown). The user can input a single target sequence or multiple sequences. When the user inputs a sequence file (‘fasta format’) containing multiple sequences, an initial alignment is produced and conserved regions are extracted as target DNA. If a single sequence is inputted it will be taken as the target DNA (Fig. 1, Green). Candidate RPA primer-probe sets are then generated. These preliminary sets undergo filtering based on user-defined parameters. If a background binding check is required a filtered set is presented in ascending order of a score that reflects the primer-probe sets ability to bind to background DNA, where smaller scores reflect a lower probability of binding. The probes are exported as raw sequences allowing the user to choose where to insert the fluorophore, dSpacer and quencher. The script guarantees the presence of two thymidine residues in the middle region of the probe for the fluorophore and quencher to be attached to.

## 3 Results

To assess the performance of *PrimedRPA*, we attempted to identify primers-probe sets in pathogens that had previously been published. For *Streptococcus pneumoniae* we used the *lepA* gene as a target (3170 bp) and for the *Bovine ephemeral fever* virus (BEFV) a terminal region in the genome (460 bp). Within 6 s, we identified 71 and 138 primers-probes sets for S. *pneumoniae* and *BEF*, respectively, including some overlapping with previously published RPA sets (Hou *et al.*, 2017; Clancy *et al.*, 2015) (Supplementary Tables S1 and S2 for parameters and output examples**)**. We also tested the software to identify primers-probes that could amplify Zika virus from any geographical region. By using 105 Zika sequences sourced globally, *PrimedRPA* identified 140 potential primer-probes sets that would bind to Zika independently of the genetic diversity. To demonstrate the utility in a setting where there is high inter-species similarities, the mitochondrial (mt) sequence (6 kb) of the *Plasmodium vivax* malaria parasite was processed with a background check using 495 mt sequences from the five other human infecting plasmodium species. Several potential sets were generated and we validated one set of primers in the laboratory (Supplementary Table S1 and File S1) that passed the background binding check. Sanger capillary sequencing confirmed that primers were specific for *P. vivax*, even in samples with mixed *P. vivax* and *P falciparum* DNA (Supplementary File S1).

## 4 Discussion

Automating the primer and exo probe design process for RPA will assist with implementing this technique and provide a stepping stone for its broader application in diagnostic tests. We have developed an *in silico* assay design tool, which provides multiple possible primers and probes that can be screened and optimized *in vitro* with the RPA technology. TwistAmp^®^ exo fluorescent probes can be converted into lateral flow probes, and therefore the *PrimedRPA* package could be used to design such applications. Further, the software can be extended as nucleic acid amplification detection kits continue to evolve and their applications in biomedical settings increase.

## Supplementary Material

Supplementary File 1

Supplementary Table 1

Supplementary Table 2
