# Supplementary material for: PrimedRPA: primer design for recombinase polymerase amplification assays
Source: Bioinformatics. 2018 Aug 8;35(4):682–4. doi: 10.1093/bioinformatics/bty701 (PMC6379019; doi:10.1093/bioinformatics/bty701)
Supplement: Supplementary File 1 [file bty701_supplementary_file_1.docx]

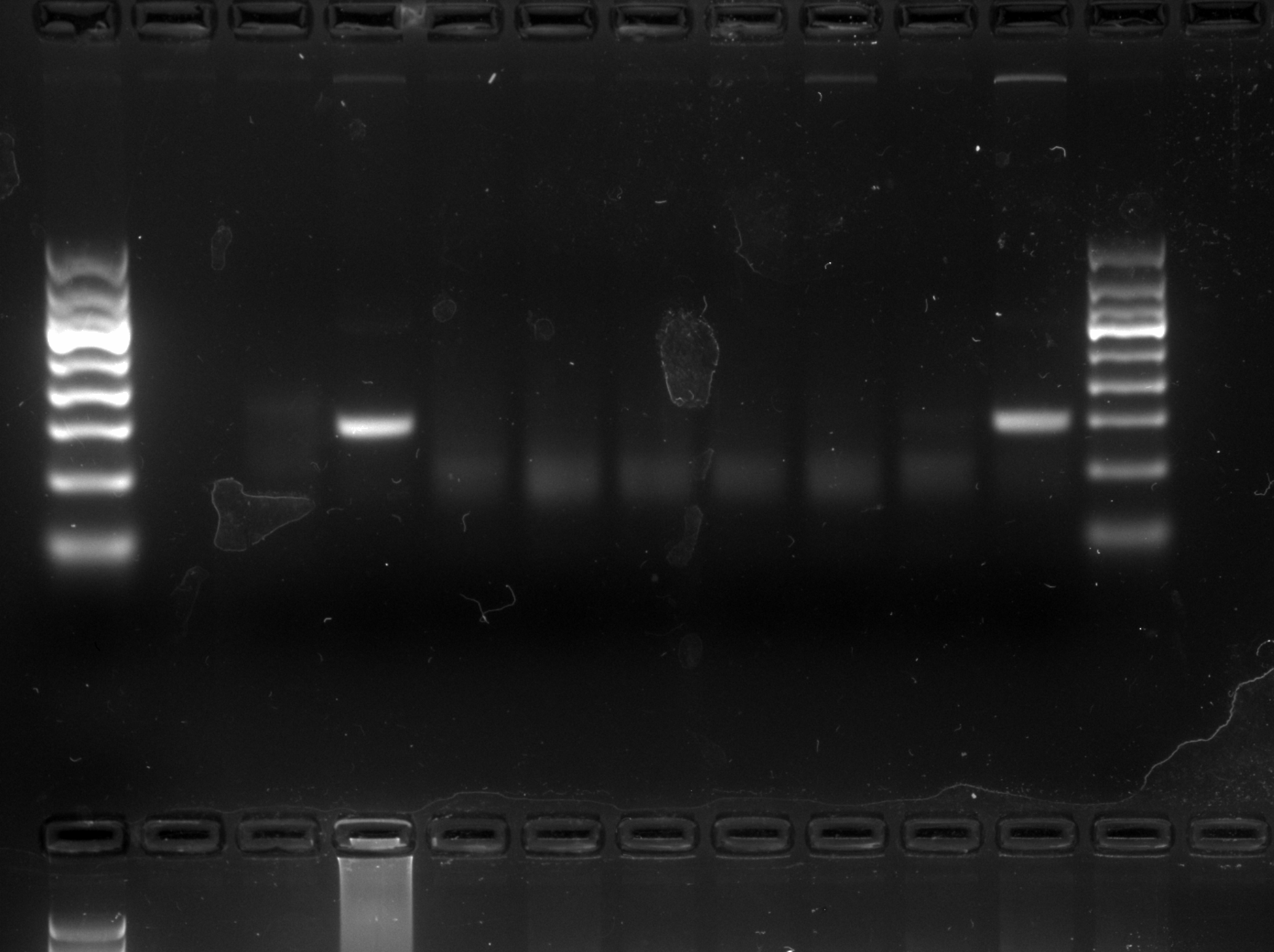


A

B

C

D

E

F

G

H

I

J

K

50bp

100bp

150bp

300bp

**A)**

**Supplementary Figure 1**. 2% agarose gel showing outcome of testing the *Plasmodium vivax* specific primers on all human infecting *Plasmodium* species. The DNA present in each well is outlined as follows: A) Qiagen GelPilot 50bp Ladder. B) Negative control (no template & no primers). C) Negative control (no template) D) *P.vivax* E) *P. falciparum* F) *P. malariae* G) *P. knowlesi* H) *P. ovale* I) *Human* J) *P. falciparum* K) *P. vivax* & *P. falciparum.*

RPA reaction was run for 30 minutes at 40^o^C. Clean up performed using SureClean Plus (Bioline).

**B) Sanger Sequencing Results ( *P. vivax* and *P. falciparum* mixed sample- K in gel)**

TTACCTAGATACTATAGTTGAACAGGACATATACATATATTCATTATTCTGAATAGAAAAAGAACTCTATAAATAACCATATAATTTCAACAAAATGCCAGTATAATATTGTAG

**PlasmoDB Blast Results for Sequence**

BLASTN 2.8.0+

Reference: Zheng Zhang, Scott Schwartz, Lukas Wagner, and

Webb Miller (2000), "A greedy algorithm for aligning DNA

sequences", J Comput Biol 2000; 7(1-2):203-14.

Reference for database indexing: Aleksandr Morgulis, George

Coulouris, Yan Raytselis, Thomas L. Madden, Richa Agarwala,

Alejandro A. Schaffer (2008), "Database Indexing for

Production MegaBLAST Searches", Bioinformatics 24:1757-1764.

RID: NHVZVZ2J014

Database: Nucleotide collection (nt)

48,886,869 sequences; 184,591,207,883 total letters

Query=

Length=114

Score E

Sequences producing significant alignments: (Bits) Value

LT635627.1 Plasmodium vivax genome assembly, organelle: mitoc... 196 8e-47

KF668406.1 Plasmodium vivax isolate 54CDC cytochrome oxidase ... 196 8e-47

JQ240416.1 Plasmodium vivax isolate V08A32 mitochondrion, com... 196 8e-47

JQ240391.1 Plasmodium vivax isolate GX5 mitochondrion, comple... 196 8e-47

JQ240387.1 Plasmodium vivax isolate GX29 mitochondrion, compl... 196 8e-47

JQ240375.1 Plasmodium vivax isolate GX15 mitochondrion, compl... 196 8e-47

JQ240368.1 Plasmodium vivax isolate GW4 mitochondrion, comple... 196 8e-47

JQ240360.1 Plasmodium vivax isolate 200667 mitochondrion, com... 196 8e-47

JQ240353.1 Plasmodium vivax isolate 200647 mitochondrion, com... 196 8e-47

JQ240351.1 Plasmodium vivax isolate 200645 mitochondrion, com... 196 8e-47

JQ240348.1 Plasmodium vivax isolate 200633 mitochondrion, com... 196 8e-47

JQ240346.1 Plasmodium vivax isolate 200629 mitochondrion, com... 196 8e-47

JQ240345.1 Plasmodium vivax isolate 200627 mitochondrion, com... 196 8e-47

JQ240334.1 Plasmodium vivax isolate 200606 mitochondrion, com... 196 8e-47

JQ240333.1 Plasmodium vivax isolate 200604 mitochondrion, com... 196 8e-47

JQ240332.1 Plasmodium vivax isolate 200603 mitochondrion, com... 196 8e-47

JQ240331.1 Plasmodium vivax isolate 200601 mitochondrion, com... 196 8e-47

KC330557.1 Plasmodium vivax isolate Lo40B cytochrome c oxidas... 196 8e-47

KC330554.1 Plasmodium vivax isolate Lo23D cytochrome c oxidas... 196 8e-47

KC330553.1 Plasmodium vivax isolate Lo23B cytochrome c oxidas... 196 8e-47

KC330550.1 Plasmodium vivax isolate Lo48A cytochrome c oxidas... 196 8e-47

KC330551.1 Plasmodium vivax isolate Lo5A cytochrome c oxidase... 196 8e-47

KC330549.1 Plasmodium vivax isolate Lo40A cytochrome c oxidas... 196 8e-47

KC330548.1 Plasmodium vivax isolate Lo8A cytochrome c oxidase... 196 8e-47

KC330547.1 Plasmodium vivax isolate Lo13C cytochrome c oxidas... 196 8e-47

KC330546.1 Plasmodium vivax isolate Lo13B cytochrome c oxidas... 196 8e-47

KC330545.1 Plasmodium vivax isolate Lo13A cytochrome c oxidas... 196 8e-47

KC330543.1 Plasmodium vivax isolate Lo1A cytochrome c oxidase... 196 8e-47

KC330542.1 Plasmodium vivax isolate Lo29C cytochrome c oxidas... 196 8e-47

KC330538.1 Plasmodium vivax isolate Lo5B cytochrome c oxidase... 196 8e-47

KC330537.1 Plasmodium vivax isolate Lo17B cytochrome c oxidas... 196 8e-47

KC330536.1 Plasmodium vivax isolate Lo17A cytochrome c oxidas... 196 8e-47

KC330535.1 Plasmodium vivax isolate Lo72A cytochrome c oxidas... 196 8e-47

KC330533.1 Plasmodium vivax isolate Lo1C cytochrome c oxidase... 196 8e-47

KC330527.1 Plasmodium vivax isolate Ca66AA cytochrome c oxida... 196 8e-47

KC330515.1 Plasmodium vivax isolate Ca60B cytochrome c oxidas... 196 8e-47

KC330513.1 Plasmodium vivax isolate Ko37A cytochrome c oxidas... 196 8e-47

KC330512.1 Plasmodium vivax isolate Ko40A cytochrome c oxidas... 196 8e-47

KC330511.1 Plasmodium vivax isolate Ko2A cytochrome c oxidase... 196 8e-47

KC330509.1 Plasmodium vivax isolate Ko35B cytochrome c oxidas... 196 8e-47

KC330508.1 Plasmodium vivax isolate Ko35A cytochrome c oxidas... 196 8e-47

KC330507.1 Plasmodium vivax isolate Ko28A cytochrome c oxidas... 196 8e-47

KC330505.1 Plasmodium vivax isolate Ko28C cytochrome c oxidas... 196 8e-47

KC330504.1 Plasmodium vivax isolate Ko9A cytochrome c oxidase... 196 8e-47

AB550280.1 Plasmodium vivax mitochondrial DNA, complete genom... 196 8e-47

AB550276.1 Plasmodium vivax mitochondrial DNA, complete genom... 196 8e-47

DQ396549.1 Plasmodium vivax isolate T9605 mitochondrion, comp... 196 8e-47

DQ396547.1 Plasmodium vivax isolate IZ01052 mitochondrion, co... 196 8e-47

AY598136.1 Plasmodium vivax isolate CX9 mitochondrion, comple... 196 8e-47

AY598135.1 Plasmodium vivax isolate CX8 mitochondrion, comple... 196 8e-47

AY598134.1 Plasmodium vivax isolate CX7 mitochondrion, comple... 196 8e-47

AY598129.1 Plasmodium vivax isolate CX2 mitochondrion, comple... 196 8e-47

AY598128.1 Plasmodium vivax isolate CX1 mitochondrion, comple... 196 8e-47

AY598121.1 Plasmodium vivax isolate VX3 mitochondrion, comple... 196 8e-47

AY598108.1 Plasmodium vivax isolate IL48 mitochondrion, compl... 196 8e-47

AY598106.1 Plasmodium vivax isolate IL45 mitochondrion, compl... 196 8e-47

AY598103.1 Plasmodium vivax isolate IBM6 mitochondrion, compl... 196 8e-47

AY598102.1 Plasmodium vivax isolate IBM5 mitochondrion, compl... 196 8e-47

AY598101.1 Plasmodium vivax isolate IBM2 mitochondrion, compl... 196 8e-47

AY598100.1 Plasmodium vivax isolate IBY7 mitochondrion, compl... 196 8e-47

AY598099.1 Plasmodium vivax isolate IBY5 mitochondrion, compl... 196 8e-47

AY598063.1 Plasmodium vivax isolate TFF13 mitochondrion, comp... 196 8e-47

AY598050.1 Plasmodium vivax isolate TC28 mitochondrion, compl... 196 8e-47

AY598039.1 Plasmodium vivax isolate T124 mitochondrion, compl... 196 8e-47

AY791690.1 Plasmodium vivax isolate pvChesson cytochrome c ox... 196 8e-47

AY791666.1 Plasmodium vivax isolate pv20131 cytochrome c oxid... 196 8e-47

AY791631.1 Plasmodium vivax isolate pv01006 cytochrome c oxid... 196 8e-47

AY791612.1 Plasmodium vivax isolate india.01018 cytochrome c ... 196 8e-47

AY791604.1 Plasmodium vivax isolate CN96 cytochrome c oxidase... 196 8e-47

AY791602.1 Plasmodium vivax isolate CN9 cytochrome c oxidase ... 196 8e-47

AY791597.1 Plasmodium vivax isolate CN78 cytochrome c oxidase... 196 8e-47

AY791596.1 Plasmodium vivax isolate CN76 cytochrome c oxidase... 196 8e-47

AY791595.1 Plasmodium vivax isolate CN75 cytochrome c oxidase... 196 8e-47

AY791593.1 Plasmodium vivax isolate CN5 cytochrome c oxidase ... 196 8e-47

AY791592.1 Plasmodium vivax isolate CN3 cytochrome c oxidase ... 196 8e-47

AY791590.1 Plasmodium vivax isolate CN12 cytochrome c oxidase... 196 8e-47

AY791589.1 Plasmodium vivax isolate CN10 cytochrome c oxidase... 196 8e-47

AY791588.1 Plasmodium vivax isolate CN1 cytochrome c oxidase ... 196 8e-47

AY791587.1 Plasmodium vivax isolate D33c cytochrome c oxidase... 196 8e-47

AY791586.1 Plasmodium vivax isolate D33b cytochrome c oxidase... 196 8e-47

AY791585.1 Plasmodium vivax isolate D33a cytochrome c oxidase... 196 8e-47

AY791583.1 Plasmodium vivax isolate Thai3 cytochrome c oxidas... 196 8e-47

AY791582.1 Plasmodium vivax isolate pv02119 cytochrome c oxid... 196 8e-47

AY791581.1 Plasmodium vivax isolate pv02087 cytochrome c oxid... 196 8e-47

AY791580.1 Plasmodium vivax isolate pv99189 cytochrome c oxid... 196 8e-47

AY791579.1 Plasmodium vivax isolate pv99174 cytochrome c oxid... 196 8e-47

AY791578.1 Plasmodium vivax isolate pv99173 cytochrome c oxid... 196 8e-47

AY791573.1 Plasmodium vivax isolate pv20196 cytochrome c oxid... 196 8e-47

AY791572.1 Plasmodium vivax isolate pv20167 cytochrome c oxid... 196 8e-47

AY791567.1 Plasmodium vivax isolate pv20041 cytochrome c oxid... 196 8e-47

AY791563.1 Plasmodium vivax isolate pv02011 cytochrome c oxid... 196 8e-47

AY791562.1 Plasmodium vivax isolate pv01157 cytochrome c oxid... 196 8e-47

AY791556.1 Plasmodium vivax isolate pvONG cytochrome c oxidas... 196 8e-47

AY791554.1 Plasmodium vivax isolate pvNorth Korean cytochrome... 196 8e-47

AY791552.1 Plasmodium vivax isolate Indonesia 14 cytochrome c... 196 8e-47

AY791528.1 Plasmodium vivax isolate pv02012 cytochrome c oxid... 196 8e-47

AY791523.1 Plasmodium vivax isolate pv205 cytochrome c oxidas... 196 8e-47

AY791522.1 Plasmodium vivax isolate pv12123 cytochrome c oxid... 196 8e-47

KY923424.1 Plasmodium vivax isolate 1089PNG mitochondrion, co... 191 4e-45

KY923423.1 Plasmodium vivax isolate 8006PNG mitochondrion, co... 191 4e-45
