# Supplementary material for: PrimedRPA: primer design for recombinase polymerase amplification assays
Source: Bioinformatics. 2018 Aug 8;35(4):682–4. doi: 10.1093/bioinformatics/bty701 (PMC6379019; doi:10.1093/bioinformatics/bty701)
Supplement: Supplementary Table 1 [file bty701_supplementary_table_1.doc]

Supplementary Table 1

**Primers designed using *PrimedRPA***

Underlined are the homologous regions between the primers designed with *PrimedRPA* and the previously published and manually designed primers; FP = Forward Primer, RP = Reverse Primer.

| **Organism** | **Sequence** |
| --- | --- |
| ***S.Pneumonia* FP** | 5′-ACAGCTCCGTCTGTTATTTACAAAGTTAATTTGAC-3′ |
| ***S.Pneumonia* RP** | 5′-AGTCCCCACGCTTACGCTGAGCTAGCTCCATTACT-3′ |
| ***S.Pneumonia* FP** | 5′-TCTGTTATTTACAAAGTTAATTTGACCGACGG-3′ |
| ***S.Pneumonia* RP** | 5′-TAGTCACAAAGTCCCCACGCTTACGCTGAGCT-3′ |
| **BEFV FP** | 5′- AGAGCTTGGTGTGAATACAGACCTTTTGTTGAC – 3′ |
| **BEFV RP** | 5′- TCGAATTTGATCAATTTTTGATAATCCTCTATC - 3′ |
| **BEFV FP** | 5′- AGCTTGGTGTGAATACAGACCTTTTGTTGACAAGAA -3′ |
| **BEFV RP** | 5′- CCTCGAATTTGATCAATTTTTGATAATCCTCTATCC -3′ |
| ***P.vivax* FP** | 5′- CCTTACGTACTCTAGCTTTTAACACAATATTATTGTC-3′ |
| ***P. vivax* RP** | 5′- ACAATATTATACTGGCATTTTGTTGAAATTATATGGT- 3′ |
